# Aging‐induced short‐chain acyl‐CoA dehydrogenase promotes age‐related hepatic steatosis by suppressing lipophagy

**DOI:** 10.1111/acel.14256

**Published:** 2024-06-19

**Authors:** Dan Deng, Shanshan Yang, Xiaoqian Yu, Ruixue Zhou, Yin Liu, Hongmei Zhang, Daxin Cui, Xingrong Feng, Yanting Wu, Xiaocun Qi, Zhiguang Su

**Affiliations:** ^1^ Molecular Medicine Research Center and National Clinical Research Center for Geriatrics, West China Hospital, and State Key Laboratory of Biotherapy Sichuan University Chengdu China

**Keywords:** acetyl‐coenzyme A, aging, autophagy, lipid droplet, lipophagy, liver steatosis, short‐chain acyl‐CoA dehydrogenase

## Abstract

Hepatic steatosis, the first step in the development of nonalcoholic fatty liver disease (NAFLD), is frequently observed in the aging population. However, the underlying molecular mechanism remains largely unknown. In this study, we first employed GSEA enrichment analysis to identify short‐chain acyl‐CoA dehydrogenase (SCAD), which participates in the mitochondrial β‐oxidation of fatty acids and may be associated with hepatic steatosis in elderly individuals. Subsequently, we examined SCAD expression and hepatic triglyceride content in various aged humans and mice and found that triglycerides were markedly increased and that SCAD was upregulated in aged livers. Our further evidence in SCAD‐ablated mice suggested that SCAD deletion was able to slow liver aging and ameliorate aging‐associated fatty liver. Examination of the molecular pathways by which the deletion of SCAD attenuates steatosis revealed that the autophagic degradation of lipid droplets, which was not detected in elderly wild‐type mice, was maintained in SCAD‐deficient old mice. This was due to the decrease in the production of acetyl‐coenzyme A (acetyl‐CoA), which is abundant in the livers of old wild‐type mice. In conclusion, our findings demonstrate that the suppression of SCAD may prevent age‐associated hepatic steatosis by promoting lipophagy and that SCAD could be a promising therapeutic target for liver aging and associated steatosis.

## Graphical abstract

Short‐chain acyl‐coenzyme A dehydrogenase (SCAD) catalyzes β‐oxidation of 4–6 carbon monocarboxylic acids in mitochondria. Here, we demonstrate that SCAD is upregulated in aged livers, and SCAD deletion is able to slow liver aging and protect the liver against age‐associated steatosis via restricting acetyl‐CoA production as well as promoting lipophagy, thereby targeting SCAD may offer therapeutic benefit for aging‐induced fatty liver.

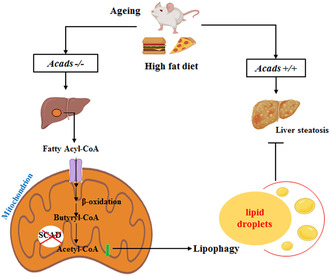

## 1 INTRODUCTION

Liver steatosis is the earliest stage of nonalcoholic fatty liver disease (NAFLD) (Chalasani et al., 2012). This disease can slowly progress to nonalcoholic steatohepatitis (NASH) with liver fibrosis and may eventually lead to more severe cirrhosis and hepatocellular carcinoma (HCC), which are associated with poor prognosis and greater mortality (Lallukka et al., 2016). In the past two decades, liver steatosis has increasingly become a common disease with a global prevalence of NAFLD. Hepatic steatosis is caused by the aberrant accumulation of triglycerides in hepatocytes due to increased fatty acid uptake from the diet and/or peripheral tissues, increased de novo lipogenesis, defects in fatty acid β‐oxidation, and reduced export to circulation in very‐low‐density lipoprotein particles (Cohen et al., 2011; Zhang et al., 2019).

Accumulating evidence highlights an elevated incidence of hepatic steatosis in elderly people (Kagansky et al., 2004). One of the earlier studies showed that the incidence of fatty liver in people older than 60 years is twofold greater than in people aged >20–40 years (Hilden et al., 1977). A more recent large cohort of type 2 diabetic outpatients also revealed that the incidence of NAFLD in persons aged ≥60 years is greater than in persons aged 40–59 years (Targher et al., 2007). Furthermore, in an Asian cohort, individuals younger than 60 years had very little severe steatosis, while those older than 60 years were more prone to severe NAFLD, such as fibrosis (Miyaaki et al., 2008). However, the mechanisms leading to the initiation and progression of hepatic steatosis in elderly individuals remain poorly understood.

Short‐chain acyl‐coenzyme A (acyl‐CoA) dehydrogenase (SCAD) is a member of the acyl‐CoA dehydrogenase family of enzymes that catalyze the first step in mitochondrial fatty acid β‐oxidation. SCAD, encoded by the *Acads* gene, targets monocarboxylic acids 4–6 carbons in length, and the favored substrate is butyryl‐CoA with four carbon units (Chen & Su, 2015). The resulting acetyl‐CoA is a crucial precursor of lipid synthesis and enters the tricarboxylic acid cycle to supply energy for tissues with high metabolic demands, such as the liver (Ghosh et al., 2016). Theoretically, SCAD deficiency limits β‐oxidation, leading to fatty acid accumulation in the liver. However, SCAD deficiency also decreases acetyl‐CoA production, which has been shown to play an important role in promoting longevity through the induction of autophagy (Bradshaw, 2021). For example, blockade of nucleocytosolic acetyl‐CoA production potentially extends *Drosophila* and yeast lifespans and clearly ameliorates age‐associated autophagy (Eisenberg et al., 2014). Furthermore, the attenuation of acetyl‐CoA synthesis in both mice and cultured human cells can induce autophagy, while high cytosolic acetyl‐CoA levels suppress autophagy and accelerate the manifestation of age‐associated pathologies (Marino et al., 2014). SCAD is considered a significant factor in age‐related disease development and progression because it functions as a metabolic modulator that controls the fate of acetyl‐CoA and rewires liver lipid metabolism.

In this study, we explored the function of SCAD in aging‐associated liver steatosis. Our findings reveal the role of SCAD and associated acetyl‐CoA in the development of fatty liver during aging. We found that SCAD ablation was able to slow liver aging and attenuate age‐related hepatic lipid accumulation. Moreover, we demonstrated that SCAD aggravated triglyceride accumulation in aged livers through an acetyl‐CoA‐impaired autophagy pathway and that SCAD deletion prevented entry into cellular senescence via the enhancement of lipophagy. This study revealed a novel role of SCAD in the hepatic aging process, and targeting SCAD may offer therapeutic benefit for aging‐induced fatty liver.

## 2 RESULTS

### 2.1 Hepatic and serum SCAD are increased in an age‐dependent manner

**FIGURE 1:**
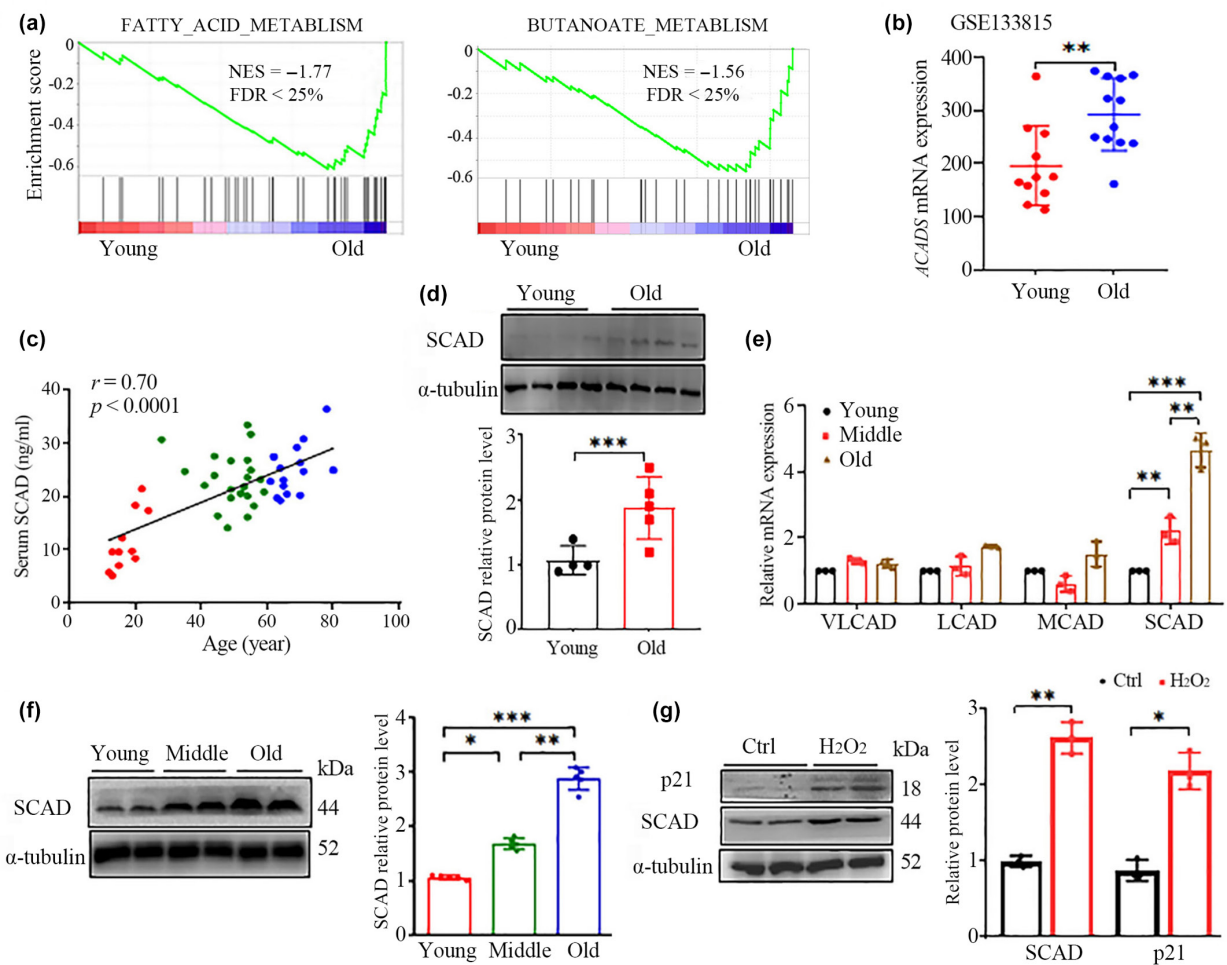
The levels of SCAD in the liver and serum increase during aging. (a, b) GSEA plots of FATTY_ACID_METABLISM and BUTANOATE_METABLISM (a) and hepatic *ACADS* mRNA expression (b) using the GSE133815 gene expression profile between 11 young (21–45 years old, male% = 54.5%) and 12 old (>69 years of age, male% = 58.3%) individuals. FDR, false discovery rate; NES, normalized enrichment score. (c) Correlation analysis of serum SCAD levels and age was conducted using the Pearson correlation test. The serum SCAD concentrations of 51 individuals with normal liver tissue were quantified via ELISA. (d) SCAD protein levels in PBMCs from normal‐liver individuals were assessed by Western blotting. (e, f) mRNA expression levels of acyl‐CoA dehydrogenase family members (VLCAD, LCAD, MCAD and SCAD) (e) and the SCAD protein levels (f) in liver tissues from wild‐type mice at the ages of 3, 10, and 20 months were quantified by real‐time PCR and Western blotting, respectively (*n* = 5 for each group). (g) The protein levels of p21 and SCAD in H_2_O_2_‐induced senescent HepG2 cells were detected by Western blotting. The values are presented as the means ± SDs of 3 independent experiments. *p < 0.05, **p < 0.01, and ***p < 0.001.

To explore the pathways associated with genes whose expression is altered by aging in the human liver, we performed an unbiased gene set enrichment analysis (GSEA) using the gene expression profiles of GSE133815, which were generated from young (21–45 years old, *n* = 11) and old (>69 years of age, *n* = 12) human liver tissues (Corton et al., 2022). All genes in GSEA were allocated into gene sets based on their known functions, and 18 gene sets were significantly enriched at nominal *p* < 0.05 and FDR *q* < 25% (Table S1). Fatty acid metabolism and butanoate metabolism were strongly enriched in the aged group (Figure 1a), suggesting that fatty acid metabolism may play a pivotal role in the pathophysiology of aging. Among the highly expressed core genes involved in the fatty acid metabolic pathway (Table S2), *ACADS*, which encodes the enzyme SCAD, is highly expressed in elderly individuals (Figure 1b).

Like those of other hepatic mitochondrial enzymes, such as glutamate dehydrogenase and malate dehydrogenase, the activity of SCAD is high in the liver, where it is localized exclusively in periportal mitochondria (Ghosh et al., 2016). Hepatic mitochondrial enzymes released into the circulation should therefore be useful for liver‐specific diagnostic markers. To further test whether SCAD is associated with aging, we measured the serum SCAD concentration in a cohort of 51 healthy liver individuals of varying ages using commercial ELISA kits. As demonstrated in Figure 1c, we detected a positive correlation between the serum SCAD concentration and age. Furthermore, because SCAD is highly expressed in peripheral blood mononuclear cells (PBMCs), which are easily accessible clinical samples and share similar gene expression patterns with other organs in the pathology of age‐related disorders (Haus et al., 2013; He et al., 2018), we determined SCAD protein levels in human PBMCs by western blotting. As expected, the level was significantly greater in the elders (over 75 years old) than in the youths (18–25 years old) (Figure 1d).

To confirm the correlation between hepatic SCAD expression and age, mice aged 3 months (young), 12 months (middle), and 24 months (old) were used to recapitulate the aging process. In addition to SCAD, the acyl‐CoA dehydrogenase family also includes members of very long‐, long‐, and medium‐chain dehydrogenases (VLCAD, LCAD, and MCAD, respectively). We first tested whether the expression levels of these genes might be associated with aging. All four transcripts were highly expressed in liver tissue, but only the expression of *Acads* mRNA increased during the aging process (Figure 1e). Accordingly, the protein content of SCAD in liver lysates determined by western blotting was significantly greater in older mice than in younger animals (Figure 1f). To validate these results in an in vitro senescence model, we used hydrogen peroxide (H_2_O_2_) to induce senescence in human HepG2 cells and mouse Hepa1‐6 hepatocytes. Compared with untreated control cells, cells treated with H_2_O_2_ exhibited classical features of senescence, as they were distinctly larger, had less proliferation, and had more positive staining for the senescence marker SA‐β‐Gal (Figure S1A). In addition, senescence was confirmed by increased expression of the cell cycle inhibitor p21 (Figure 1g and Figure S1B). As expected, H_2_O_2_‐induced senescent hepatocytes expressed more SCAD protein than did non‐senescent cells (Figure 1g and Figure S1B). Collectively, these findings confirmed a positive correlation between SCAD concentration and age.

### 2.2 SCAD levels are positively associated with age‐related liver steatosis

**FIGURE 2:**
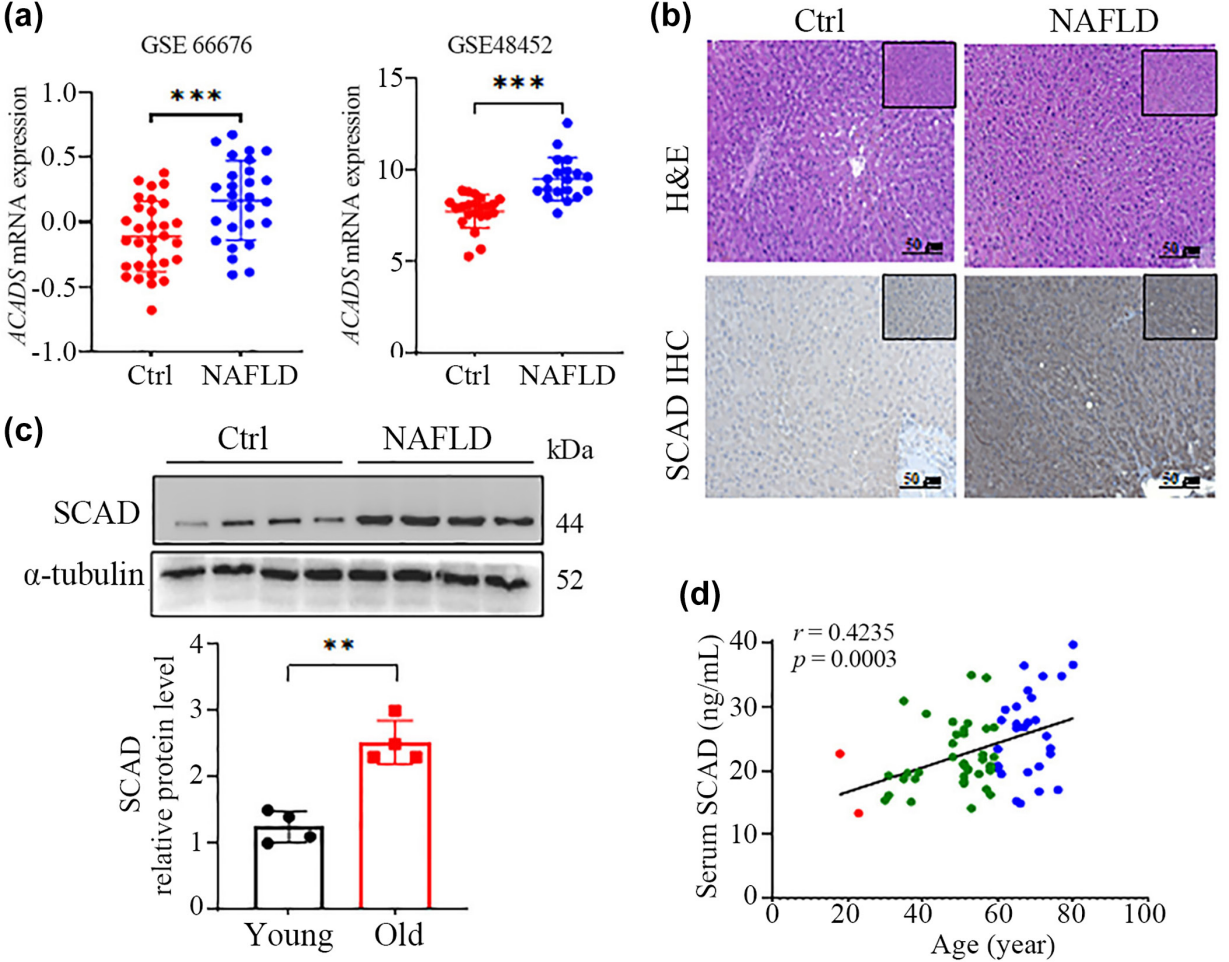
SCAD levels are positively associated with age‐related liver steatosis. (a) Hepatic *ACADS* mRNA expression in normal controls and NAFLD patients from the GEO datasets GSE66676 (control: *n* = 34, male% = 17.6%; NAFLD: *n* = 26, male% = 23.1%) (left) and GSE48452 (control: *n* = 27, male% = 7.4%; NAFLD: *n* = 14, male% = 41.7%) (right). (b) Representative H&E staining (upper) and SCAD IHC staining (lower) of liver sections from NAFLD patients and healthy individuals. (c) SCAD protein levels in PBMCs from NAFLD patients (*n* = 4) and age‐matched healthy controls (*n* = 4) were assessed via Western blotting. (d) Correlation analysis of serum SCAD levels and age was conducted using the Pearson correlation test. Serum SCAD concentrations were measured by ELISA in 68 NAFLD patients at various ages. *p < 0.05, **p < 0.01, and ***p < 0.001.

SCAD levels increase in the liver during aging, and growing evidence suggests that NAFLD incidence is markedly increased in aging humans. Thus, we wanted to determine whether SCAD could correlate with the age‐related occurrence of hepatic steatosis. To this end, we first evaluated *ACADS* mRNA expression in the GEO datasets GSE66676 and GSE48452, which represent hepatic gene expression in both adolescent (GSE66676, control = 34, NAFLD = 33) and aged (GSE48452, control = 45, NAFLD = 18) normal controls and NAFLD patients, respectively (Ahrens et al., 2013; Xanthakos et al., 2015). *ACADS* mRNA expression was distinctly upregulated in NAFLD livers compared with that in normal livers (Figure 2a). To confirm the upregulation of hepatic SCAD in NAFLD, we determined SCAD protein levels in human liver tissues by IHC staining, and the quantitative results showed that the amount of SCAD protein was significantly greater in the livers of NAFLD patients (Figure 2b). Moreover, the level of the SCAD protein in PBMCs was also markedly greater in NAFLD patients than in age‐matched healthy controls (Figure 2c). Subsequently, the serum SCAD concentrations were measured in 68 NAFLD patients at various ages (Table S1), and a significant positive correlation was observed between the serum SCAD concentration and age (Figure 2d). Collectively, these observations indicated that the age‐dependent upregulation of SCAD was correlated with the development of NAFLD during aging.

### 2.3 SCAD depletion protects mice against age‐related liver pathological damage

**FIGURE 3:**
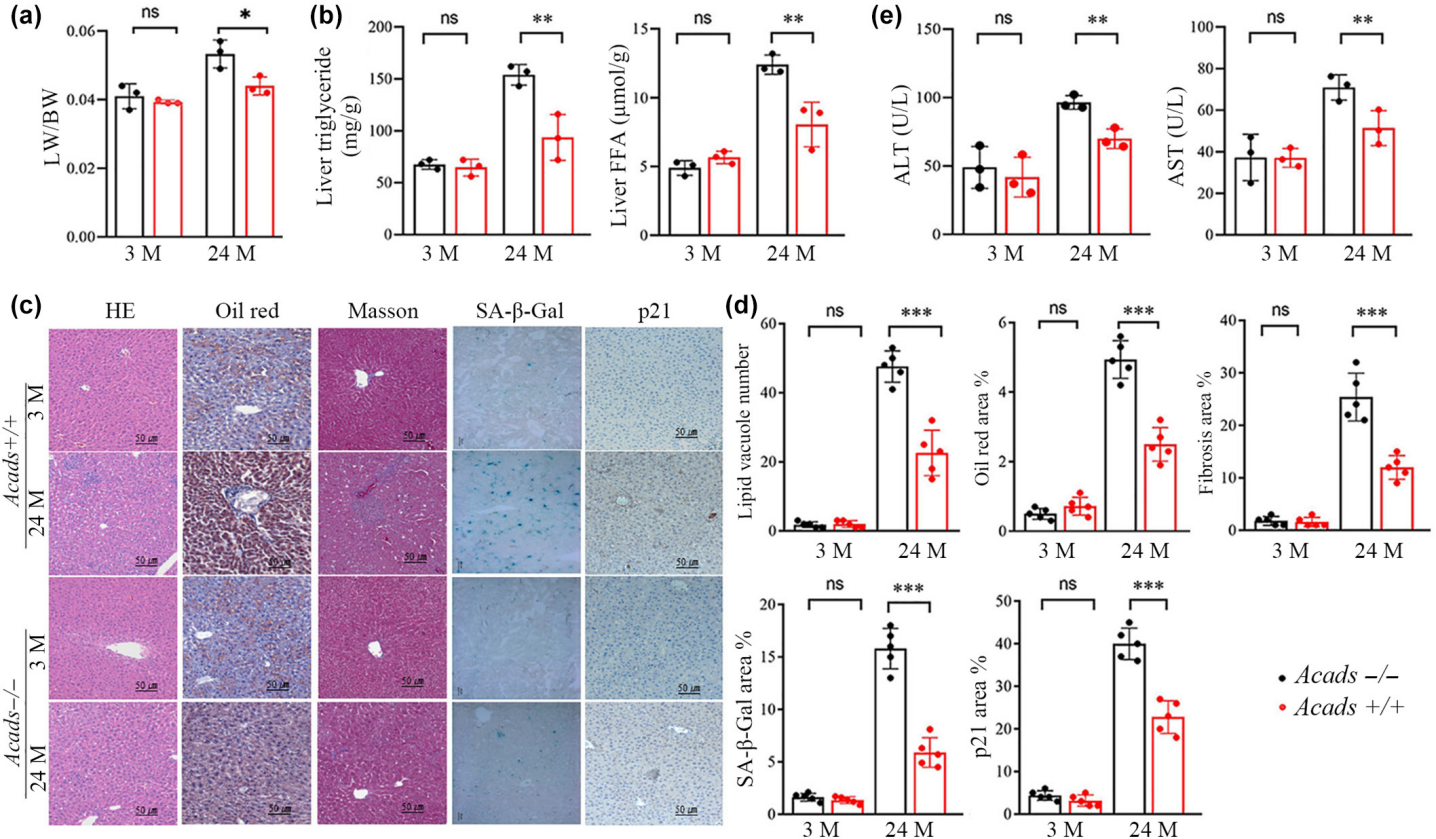
SCAD depletion protects mice against age‐related liver pathological damage. *Acads+/+ m*ice and *Acads−/−* mice were fed a chow diet for 3 months (3 M) or 24 months (24 M). (a) Liver/body weight (LW/BW) ratio. (b) Hepatic triglyceride and fatty acid (FA) concentrations. (c) Representative images of liver sections stained with H&E, Oil Red O, Masson's trichrome, senescence‐associated (SA)‐β‐galactosidase, or p21 antibody (scale bar = 50 μm). (d) Quantitative analysis of the number of lipid vacuoles in the liver and the areas of Oil Red O‐, Masson's trichrome‐, β‐galactosidase‐, or p21‐positive staining. Three micrographs of each mouse were used for quantification (*n* = 5). (e) Serum ALT and AST concentrations (*n* = 3). The data are presented as the mean ± SD. *p < 0.05, **p < 0.01, and ***p < 0.001.

The BALB/cByJ strain (*Acads −*/−) derived from the BALB/cJ strain (*Acads +*/+) is deficient in the SCAD enzyme due to a spontaneous mutation that results in a 278‐bp deletion at the 3′ end of the *Acads* gene (Chen et al., 2019; Su et al., 2010). Western blotting analysis of liver tissues from BALB/cBy mice revealed that the SCAD protein was completely deficient (Figure S2A). Although the body weights of the *Acads−*/− mice and their age‐ and sex‐matched *Acads+*/+ controls were comparable (Figure S2B), the liver‐to‐body weight ratio was significantly lower in the old *Acads−*/− mice than in the old *Acads+*/+ mice (Figure 3a). Lipid analysis of liver tissue and serum revealed that triglyceride (TG) and fatty acid (FA) levels were comparable between *Acads−*/− mice and *Acads+*/+ controls at 3 months of age, while aged (24‐month‐old) *Acads*−/− mice presented significantly lower concentrations of TG and FA than did age‐matched control mice (Figure 3b, Figure S2C,D). Similarly, H&E and Oil Red O staining of liver sections revealed that young animals had barely detectable hepatosteatosis; on the other hand, aged control mice exhibited distinct accumulation of lipid droplets and steatosis, which was ameliorated in aged *Acads−*/− mice (Figure 3c,d). In addition, the changes in other aging‐associated liver damage markers, such as elevated aminotransaminases (AST and ALT), were significantly mitigated by SCAD deficiency (Figure 3e). Strikingly, *Acads* deficiency attenuated fibrosis development in the aged livers of old mice (Figure 3c,d). Considering that senescence is a hallmark of aging and that senescent cells can contribute to age‐related hepatic lipid accumulation, we also measured the senescence markers SA‐β‐Gal and p21 in liver tissues. Compared to those of age‐matched control mice, the livers of old *Acads−*/− mice displayed a senescence‐resistant phenotype with significantly fewer SA‐β‐Gal‐positive cells and lower p21 levels (Figure 3c,d). Taken together, these data provide evidence that SCAD deficiency can delay liver aging and suppress age‐related liver steatosis.

### 2.4 SCAD inhibits autophagy in the liver during aging

Next, we utilized our previous whole‐transcriptome data (GSE128608) of liver tissues from *Acads+*/+ and *Acads−*/−mice fed a high‐fat diet (Chen et al., 2019) to identify the possible mechanisms by which *Acads* ablation protects against liver aging. A high‐fat diet (HFD) induced significant hepatic steatosis, as indicated by H&E and Oil Red O staining (Figure S3). Given that hepatic fat accumulation can trigger oxidative stress and that the latter is regarded as the main cause of aging (de Almeida et al., 2022), we speculated that a HFD could induce liver aging. Indeed, a HFD increased senescence‐associated (SA)‐β‐galactosidase activity and the expression of the cell cycle inhibitor p21 (Figure S3). *Acads* ablation significantly ameliorated HFD‐induced hepatic lipid accumulation and liver aging, as indicated by the reductions in SA‐β‐gal activity and p21 levels (Figure S3).

**FIGURE 4:**
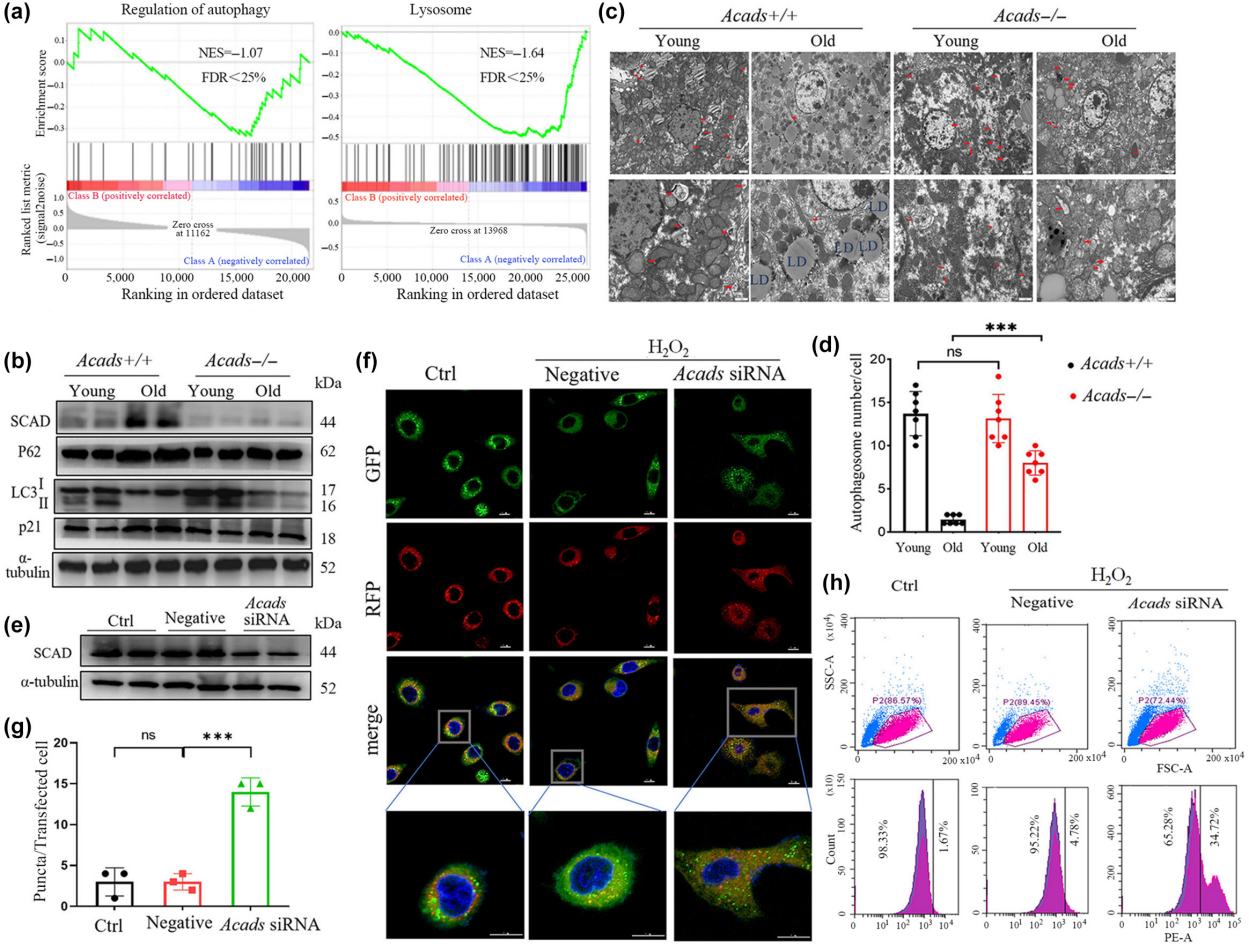
SCAD inhibits autophagy in the liver during aging. (a) GSEA was conducted by examining the transcriptome (GSE128608) of mice with an *Acads+/+* or *Acads−/−* genetic background, and enrichment plots showing the regulation of autophagy and lysosomal pathways were generated. NES, normalized enrichment score; FDR, false discovery rate. (b–d) Hepatic protein levels of autophagy markers (p62, LC3 and p21) were measured by Western blotting (b) and autolysosome ultrastructural features were assessed by TEM (c) in young (3 M) and old (24 M) mice with an *Acads+/+* or *Acads−/−* genetic background. The overall average number of autophagosomes in both genotypes was determined from 8 randomly selected fields (d). (e–h) HepG2 cells were transfected with *Acads* siRNA for 2 days and then treated with 300 μM H_2_O_2_ for 4 days. Autophagic flux was assessed with a dual tandem‐tagged GFP‐RFP‐LC3 adenovirus. (e) Western blot analysis of the effect of *Acads* siRNA on SCAD protein levels. (f) Images of representative HepG2‐LC3 cells (scale bar: 10 μm). (g) Quantification of LC3 puncta in HepG2‐LC3 cells in at least three independent experiments. (h) HepG2‐LC3 cells were analyzed by flow cytometry. *p < 0.05, **p < 0.01, and ***p < 0.001.

Initially, we identified 117 differentially expressed genes (DEGs) between *Acads+*/+ and *Acads−*/− mice. We then characterized the functional roles and biological processes of this gene set using unbiased GSEA. The results showed that 11 signaling pathways were highly enriched, with nominal *p* < 0.05 and FDR *q* < 25% (Table S3), and “lysosome” and “regulation of autophagy” were the main pathways associated with low expression of *Acads* (Figure 4a, Table S4). Thereafter, we verified whether SCAD deficiency could induce autophagy in aged livers. As a result, the conversion of LC3‐I to LC3‐II, a well‐accepted biomarker of autophagic activity, was markedly reduced in the livers of old mice of both genotypes, but the old *Acads−*/− mice maintained a greater level of LC3‐II protein than did the old *Acads+*/+ controls (Figure 4b). Moreover, we found that the levels of the p62 protein, which mediates LC3 ubiquitination and degradation, were comparable between the livers of old *Acads−*/− mice and young animals and were significantly greater in aged wild‐type mice (Figure 4b). Additionally, we examined autophagy by TEM, and the results indicated that the number of autophagosomes was large and undistinguishable in young mice of both genotypes. The old *Acads−*/− mice exhibited an approximately 39% reduction in the number of autophagosomes, which were barely detected in the livers of the old wild‐type mice (Figure 4c,d). These data revealed that SCAD deficiency might contribute to the maintenance of autophagy in the aged liver.

To further confirm the impact of SCAD on autophagy during aging, we examined autophagic flux in H_2_O_2_‐induced senescent HepG2 cells in which SCAD was knocked down by siRNA (Figure 4e). The cells were transfected with tandem fluorescent‐tagged RFP‐GFP‐LC3 adenovirus, which enables the detection of autophagic flux by monitoring green GFP‐LC3 and/or red RFP‐LC3 puncta (Yu et al., 2023). Our data showed that H_2_O_2_ treatment significantly blocked autophagic flux, as indicated by the presence of more autophagosomes with fewer autolysosomes, which are indicated by yellow puncta and red puncta, respectively, in the merged image. In contrast, SCAD knockdown resulted in fewer early autophagosomes with more autolysosomes (Figure 4f,g). These findings further validated by FACS analysis that the inhibition of *Acads* expression could induce the consumption of autophagosomes and further enhance autophagic flux in senescent cells (Figure 4h).

### 2.5 SCAD exacerbates senescence‐associated liver steatosis through the acetyl‐coenzyme A‐dependent lipophagy pathway

**FIGURE 5:**
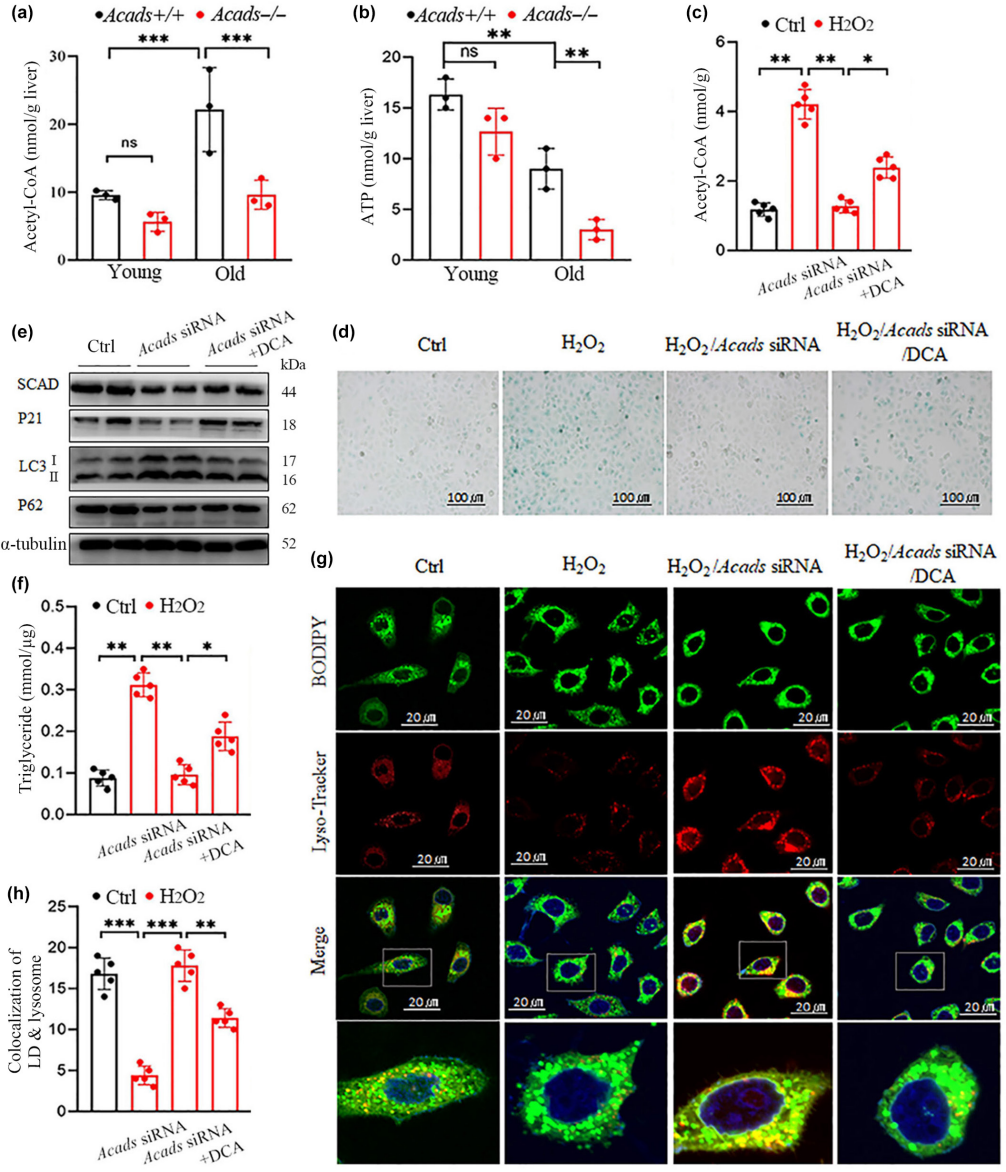
SCAD depletion alleviates senescence‐associated liver steatosis through the acetyl‐coenzyme A‐dependent lipophagy pathway. (a, b) Hepatic acetyl‐CoA concentrations (a) and ATP production (b) were quantified via ELISA in 3‐month‐old (young) and 24‐month‐old (old) *Acads+/+* or *Acads−/−* mice (*n* = 3 for each group). (c–h) Senescence was induced in HepG2 cells with or without *Acads* knockdown by *Acads* siRNA via treatment with 300 μM H_2_O_2_ for 4 days; thereafter, the cells were treated with 12 μM dichloroacetic acid (DCA) for 24 h. (c) Cellular acetyl‐CoA production, (d) SA‐β‐galactosidase staining (bar scale: 100 μm), (e) immunoblots of SCAD, p21, LC3I/LC3II and p62, (f) cellular triglyceride content, (g) representative immunofluorescence images of colocalization between lysosomes labeled with LysoTracker Red and lipid droplets labeled with the green lipid dye BODIPY (bar scale: 20 μm), (h) bar graphs showing the percentage of colocalization between lysosomes and lipid droplets. *p < 0.05, **p < 0.01, and ***p < 0.001.

Autophagy is a critical and fine‐tuned process for maintaining energy homeostasis. Given that acetyl‐CoA is a phylogenetically conserved inhibitor of age‐associated autophagy and that adenosine triphosphate (ATP) is regarded as a reservoir of energy for the lifespan (Eisenberg et al., 2014), we therefore determined the hepatic acetyl‐CoA concentration and ATP production. The results showed that the acetyl‐CoA concentration increased while ATP production decreased during aging and that the loss of *Acads* resulted in a dramatic decrease in the levels of both acetyl‐CoA and ATP in old mice (Figure 5a,b). These findings suggest that SCAD inhibits autophagy to induce hepatic lipid accumulation during aging through an acetyl‐CoA‐dependent pathway.

Subsequently, we explored whether acetyl‐CoA is necessary for the inhibition of autophagy by which SCAD modulates hepatocyte senescence and lipid accumulation in the liver. With or without *Acads* knockdown by *Acads* siRNA, senescence was induced in HepG2 cells treated with 300 μM H_2_O_2_ for 4 days; thereafter, the cells were treated with 12 μM DCA dichloroacetic acid (DCA), which increases cellular acetyl‐CoA levels by suppressing pyruvate dehydrogenase kinase (PDK) and subsequently increasing the activity of pyruvate dehydrogenase (PDH) (Zhou et al., 2001). H_2_O_2_ treatment induced significant increases in acetyl‐CoA production and SA‐β‐gal activity, which were sharply reduced by *Acads* ablation (Figure 5c,d). Moreover, the suppression of acetyl‐CoA production by *Acads* siRNA was significantly blunted by DCA in HepG2 cells (Figure 5c). Correspondingly, DCA almost completely abolished the protective effect of *Acads* knockdown on senescence in HepG2 cells, as indicated by the significant increase in SA‐β‐gal activity (Figure 5d) and p21 expression (Figure 5e). In line with these observations, DCA reversed the increase in LC3II/LC3I and the decrease in p62 caused by *Acads* siRNA in H_2_O_2_‐challenged HepG2 cells (Figure 5e), indicating the inhibition of autophagy. Furthermore, quantification of the intracellular triglyceride levels revealed that *Acads* siRNA significantly decreased the triglyceride content in H_2_O_2_‐treated cells, which was effectively blocked by DCA (Figure 5f). Since triglycerides are the major component of lipid droplets (LDs), their content in treated cells was also evaluated. BODIPY staining data showed that DCA significantly attenuated the inhibitory effects of *Acads* ablation on H_2_O_2_‐induced LD accumulation (Figure 5g). Given that lipophagy, or autophagic degradation of LDs, is one of the major mechanisms involved in maintaining LD homeostasis in hepatocytes (Scorletti & Carr, 2022), we examined the involvement of SCAD and its associated acetyl‐CoA in the modulation of lipophagy. In H_2_O_2_‐induced senescent HepG2 cells, the colocalization of LysoTracker and BODIPY revealed that *Acads* siRNA effectively induced the capture of LDs by autolysosomes, while this effect was suppressed by DCA treatment (Figure 5g,h), suggesting that SCAD could impair lipophagy in an acetyl‐CoA‐dependent manner under senescent conditions.

To further confirm the effect of SCAD expression on the lipophagic pathway, we infused an adenovirus‐encoding SCAD (Ad‐*Acads*) into HepG2 hepatocytes (Figure S4A). As expected, Ad‐*Acads*‐infused hepatocytes exhibited markedly increased acetyl‐CoA levels (Figure S4B) and p62 expression but decreased LC3II levels (Figure S4A). Moreover, the number of LDs that colocalized with autolysosomes was greatly reduced by Ad‐*Acads* infusion (Figure S4C,D). In addition, more triglycerides accumulated in Ad‐*Acads*‐infused hepatocytes (Figure S4E). Correspondingly, the infusion of Ad‐*Acads* significantly induced hepatocyte senescence, as reflected by the accumulation of SA‐β‐gal (Figure S4F) and p21 (S4A). Taken together, these data confirmed that SCAD exacerbates senescence‐associated liver steatosis through the acetyl‐CoA‐dependent autophagy pathway.

### 2.6 SCAD depletion alleviates replicative senescence

**FIGURE 6:**
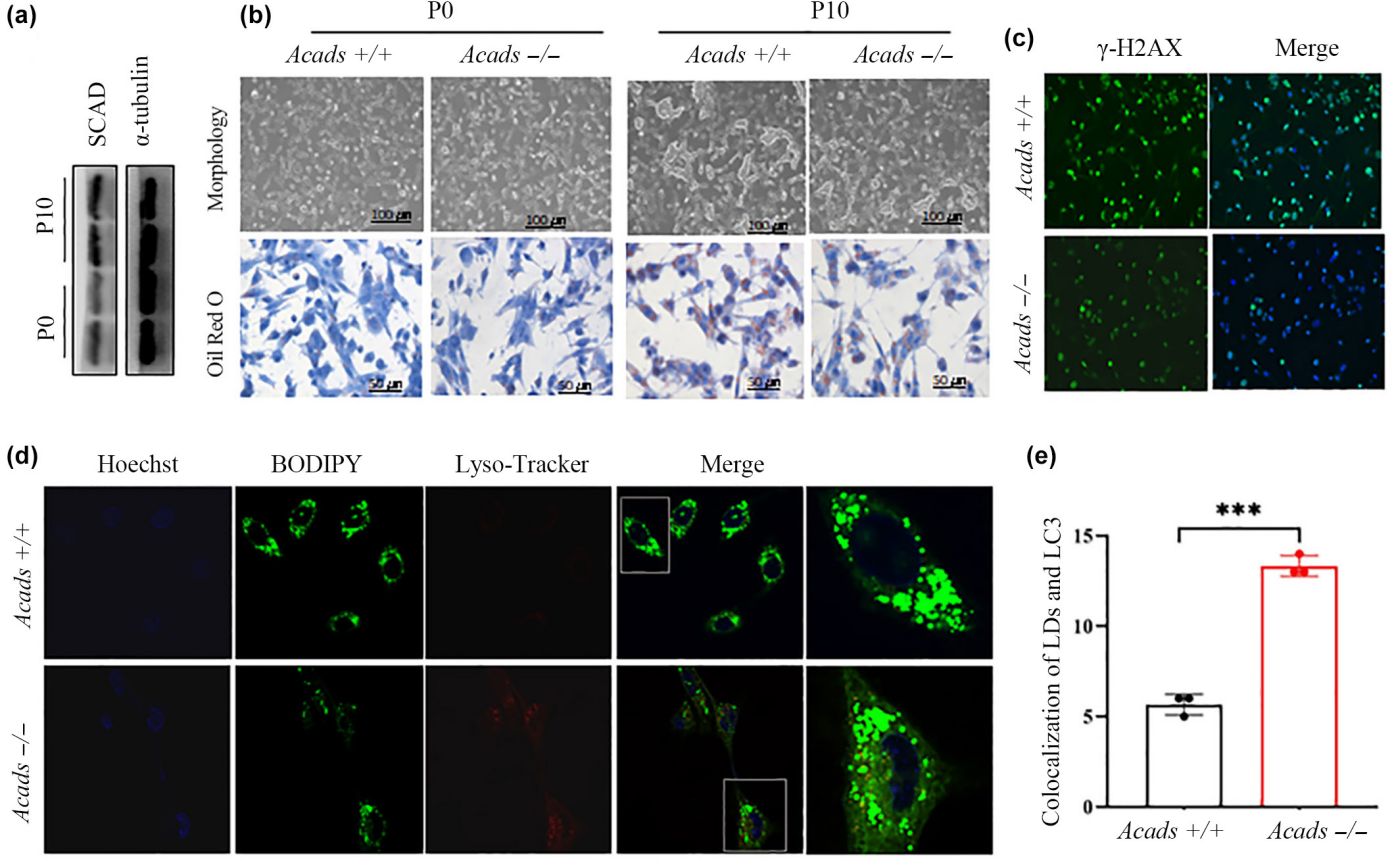
SCAD ablation protects cells against replicative senescence. Primary MEFs with an *Acads+/+* or *Acads−/−* genetic background were prepared from mouse embryos on embryonic day 14 and cultured in DMEM for 2 days; these cells were labeled passage 0 (P0). Thereafter, the MEFs were serially cultured to passage 10 (P10) to establish replicative senescent cells. (a) SCAD protein levels were assessed in primary MEFs at P0 and P10 by Western blotting. (b) Cellular morphology was monitored with a light microscope (upper, scale bar: 100 μm), and lipid accumulation in cells was assessed by Oil Red O staining (lower, scale bar: 50 μm). (c) γ‐H2AX content in the P10 MEFs was examined via immunofluorescence staining. (d) Representative immunofluorescence images showing the colocalization of lysosomes labeled with LysoTracker Red and lipid droplets labeled with the green lipid dye BODIPY (scale bar: 50 μm). (e) Bar graphs showing the percentage of colocalization between lysosomes and lipid droplets. ***p < 0.001.

We determined whether acetyl‐CoA‐induced lipophagy contributes to replicative senescence during serial passaging. Primary mouse embryonic fibroblasts (MEFs) with limited proliferative capacity are widely used as a replicative senescence model. The protein level of SCAD in the cell lysates was significantly greater in the P10 MEFs than in the very early passage MEFs (P0) (Figure 6a). Furthermore, the morphology and storage of lipids were distinct between the P10 MEFs and control P0 cells. By passage 10 (P10), the *Acads*+/+ MEFs exhibited distinct morphologies of senescent cells with enlarged and flattened shapes, which was not obvious in the *Acads*‐deficient MEFs (Figure 6b). Similarly, cellular lipid storage was also significantly lower in the *Acads−/−* MEFs than in the *Acads*+/+ controls (Figure 6b), consistent with the decrease in senescence detected in *Acads*‐deficient cells. Cellular senescence can be triggered by the accumulation of irreparable DNA damage, which induces H2AX phosphorylation (denoted as γH2AX) at the region of DNA double‐strand breaks. Thus, we assessed the levels of γH2AX by immunofluorescence staining. Compared to those in *Acads*+/+ mice, P10 MEFs derived from *Acads−/−* mice exhibited significant inhibition of the DNA damage response, as visualized by a decreased γH2AX signal compared to that in control cells (Figure 6c). Staining of lipid droplets and autolysosomes with BODIPY and LysoTracker, respectively, revealed that the number of autolysosomes that captured lipid droplets was markedly greater in *Acads*‐deficient MEFs than in *Acads*+/+ MEFs at passage 10 (Figure 6d,e). Thus, our results confirmed that SCAD ablation prevents cellular senescence by enhancing lipophagy during replicative senescence.

## 3 DISCUSSION

Aging is a complex multifaceted process in which metabolism plays a significant role. The liver is an essential metabolic organ that maintains metabolic homeostasis to meet body energy requirements. Epidemiological studies have demonstrated that hepatic steatosis is one of the main characteristics of an aging liver (Bertolotti et al., 2014) despite a lower prevalence of fatty liver in some very old age groups (Sheedfar et al., 2013). Although several pathways, such as endoplasmic reticulum stress, deregulated nutrient sensing, mitochondrial dysfunction, and cellular senescence pathways, have been implicated in age‐associated hepatic steatosis (Cui et al., 2024; Hunt et al., 2019), the molecular mechanism underlying aging‐related impairments in hepatic lipid homeostasis remains elusive.

In this study, we first demonstrated that SCAD is a key regulator of aging‐associated hepatic steatosis. Our findings showed that hepatic SCAD is upregulated in both aging humans and mice. In agreement with these results, knockdown of SCAD protected mice against age‐related liver pathological damage. At the molecular level, intrahepatic acetyl‐CoA, a product of the SCAD enzyme, markedly accumulated during the aging process. Further evidence suggested that SCAD aggravated triglyceride accumulation in aged livers through the acetyl‐CoA‐dependent autophagy pathway and that SCAD ablation prevented entry into cellular senescence via the enhancement of lipophagy.

SCAD deficiency blocks the mitochondrial fatty acid β‐oxidation of C4–C2, resulting in the accumulation of butyric acylcarnitine and a reduction in acetyl‐CoA. Acetyl‐CoA is not only a key metabolite that bridges fatty acid β‐oxidation and de novo lipogenesis as well as glycolysis and the tricarboxylic acid cycle but also a substrate of histone acetyltransferases regulating memory formation (Mufson et al., 2008). The amount of acetyl‐CoA in young individuals is much lower than that in elderly individuals because acetyl‐CoA can be complemented by ATP citrate lyase (ACLY), whose expression in aged liver or adipose tissues is markedly inhibited (Carrer et al., 2017). Although some studies suggest that an increase in acetyl‐CoA levels can increase the acetylation of histone 3 at lysine 9 (H3K9) to reduce brain aging (Currais et al., 2019), there is evidence that decreased acetyl‐CoA during *C. elegans* development is required for the mitochondrial unfolded protein response (UPR^mt^) to extend lifespan (Zhu et al., 2020). Cytoplasmic acetyl‐CoA clearly plays a pro‐aging role in many tissues through the inhibition of autophagy, and caloric restriction decreases acetyl‐CoA levels to stimulate longevity‐promoting autophagy by restraining the activity of p300 histone acetyltransferase (Hagopian, 2003). Furthermore, this is corroborated by the fact that loss of the histone acetyltransferase NAA40 resulted in hepatic lipid accumulation by increasing acetyl‐CoA levels (Charidemou et al., 2022). Taken together, our results suggest that the mitochondrial‐derived pool of acetyl‐CoA can inhibit autophagy, attenuating the autophagic degradation of lipid droplets in hepatocytes. Indeed, nutrient deprivation‐induced reductions in acetyl‐CoA levels are sufficient to induce autophagy (Marino et al., 2014). Although SCAD inactivation limits fatty acid β‐oxidation, the decrease in acetyl‐CoA production relieves the suppression of autophagy, which overcomes hepatic lipid overload. This might explain why SCAD inactivation can protect against hepatic steatosis during aging.

Lipophagy is a specific autophagy pathway that selectively removes excess lipids to maintain lipid homeostasis and is also thought to contribute substantially to the hydrolysis of triglycerides in cells (Cingolani & Czaja, 2016). Hence, promoting lipophagy might be an effective strategy for ameliorating hepatic steatosis (Schulze et al., 2017). However, the underlying contribution of lipophagy to lipolysis remains unclear. In a study that initially identified the autophagy chaperone p62/SQSTM1 as an essential adaptor for discerning and delivering specific protein aggregates to autophagosomes for degradation (Jeong et al., 2019). Generally, most studies have shown that p62 is linked to the formation of ubiquitin‐positive inclusions and binds LC3II to facilitate autophagic degradation, but not all of these interactions (Nihira et al., 2014), which means that the inhibition of hepatic autophagy promotes steatosis. Crosstalk between lipophagy and p62‐mediated ubiquitin lipolysis occurs (Liu et al., 2016), and it is possible that activation of lipophagy results in protection against hepatic lipid accumulation under certain metabolic conditions (Robichaud et al., 2021). In line with these studies, our results suggest that *Acads* deficiency results in the activation of p62‐mediated autophagic degradation of lipids in mice to promote aging‐ or HFD‐induced fatty liver.

Collectively, these studies identified a previously unrecognized mitochondrial–lysosomal metabolic link that promotes the autophagic degradation of lipid droplets. Our results revealed that β‐oxidation of fatty acids is a major source of acetyl‐CoA that maintains hepatic lipid homeostasis during aging. Induction of lipid autophagy via inhibition of acetyl‐CoA production might be a promising therapeutic option for aging‐associated fatty liver disease.

## 4 EXPERIMENTAL PROCEDURES

### 4.1 Data sources and gene set enrichment analysis (GSEA)

Gene Expression Omnibus (GEO) datasets (GES133815, GSE66676, and GSE48452) representing human hepatic gene expression in both young and old individuals with or without NAFLD were downloaded from the National Center for Biotechnology Information (NCBI) website (https://www.ncbi.nlm.nih.gov/geo/). Our previously available raw transcriptome data (GSE128608) were generated from mouse liver tissues.

GSEA 4.1.0 software (https://www.gsea‐msigdb.org/gsea/index.jsp) was used to enrich pathway‐associated genes whose expression was altered either by aging in human liver tissues or by *Acads* expression in mouse liver tissues. Gene expression profiles of GSE133815 or GSE128608 were imported into the GSEA desk application according to the instructions, and “C2.cp.KEGG.v7.2.symbols” from the Molecular Signatures Database (MSigDB) was used as a reference gene set. Using a permutation test with 1000 repetitions, enriched gene sets with a nominal *p* value of <0.05 and a false discovery rate (FDR) of <0.25 were considered to be significantly different, but any gene sets that had <10 genes and >500 genes were excluded to avoid inflated scores for small gene sets and inaccurate normalization for larger gene sets. Genes were ranked based on their magnitude of difference in expression between the phenotypic states.

### 4.2 Patients

This study was approved by the Institutional Review Board of West China Hospital. The patients provided informed consent prior to participation. This study included 68 individuals with a clinical diagnosis of NAFLD according to the Kleiner criteria and 51 healthy controls (Table S5). All the subjects signed a written consent form.

### 4.3 Animals

All animal care and experiments were conducted in accordance with a protocol approved by the Animal Care and Use Committee of West China Hospital of Sichuan University. BALB/cBy and BALB/cJ mice were purchased from The Jackson Laboratory (Bar Harbor, ME, USA). Mice were housed with free access to food and water on a 12‐h light/dark cycle. Mice were randomly divided into three groups: young (3 months old), middle (12 months old), and old (24 months old) on a standard chow diet.

### 4.4 Reagents and plasmids

DMEM/F12 and Hieff Trans® liposomal transfection reagents were purchased from YSASEN Biotech (Shanghai, China). Oil Red O (HY‐D1168), BODIPY 493/503 (HY‐W090090), and LysoTracker Red (HY‐D1300) were purchased from MedChemExpress. An ATP assay kit (S0026), an SA‐β‐gal staining kit (C0602), and a tandem‐tagged GFP‐RFP‐LC3 adenovirus (C3011) were purchased from Beyotime Biotech (Shanghai, China). Adenovirus‐encoding SCAD (Ad‐*Acads*) and small interfering RNA (siRNA) targeting *Acads* and scramble control siRNA were purchased from Sangon Biotech (Shanghai, China). ELISA kits for human SCAD (JM‐1489H2) and mouse acetyl‐CoA (ml037242) were purchased from Jiangsu Jingmei Biotech (www.jsjmsw.com) (Yancheng, Jiangsu, China) and Shanghai Enzyme‐linked Biotech (www.mlbio.cn) (Shanghai, China), respectively. Quantitative kits for triglyceride (A110‐1‐1), total cholesterol (A111‐1‐1), alanine transaminase (ALT, C009‐2‐1), and aspartate transaminase (AST, C010‐2‐1) were purchased from Nanjing Jiancheng Bioengineering Institute (Nanjing, China). A total protein extraction kit (KGB5303) and bicinchoninic acid (BCA) protein assay kit (KGB2101) were purchased from Jiangsu KeyGen BioTech (Nanjing, China). TRIzol reagent was purchased from Invitrogen (Carlsbad, CA), and a cDNA synthesis kit (R312) and SYBR qPCR Master Mix (Q511) were purchased from Vazyme Biotech (Nanjing, China). Antibodies against β‐actin (1:3000, M1501), p62 (1:1000, ET1603‐34), and LC3 (1:2000, ET1603‐11) were purchased from HuaBio (Hangzhou, China). Antibodies against SCAD (1:1000; ab156571) and γ‐H2A.X (ab2893) were purchased from Abcam (Shanghai, China). Enhanced chemiluminescence (ECL) reagents were purchased from ComWin Biotech (Beijing, China).

### 4.5 Cell culture and senescence induction

HepG2 and Hepa1‐6 hepatocyte cells (ATCC) were cultured at 37°C in a humidified 5% CO_2_ atmosphere in DMEM supplemented with 10% (v/v) heat‐inactivated fetal bovine serum (FBS), 1% antibiotics (100 U/mL penicillin and 100 μg/mL streptomycin), 1% glutamine, and 0.01% nonessential amino acids (Irvine et al., 2014). To induce senescence, cells were treated with various concentrations of H_2_O_2_ in DMEM for four consecutive days, and the optimal conditions (0.3 mM for HepG2 cells and 0.4 mM for Hepa1‐6 cells) for the induction of senescence rather than apoptosis or death were determined.

Mouse embryonic fibroblasts (MEFs) were prepared from embryos on embryonic day 14 and grown in DMEM supplemented with 10% (v/v) FBS and 1% antibiotics for 2 days; thereafter, the cells were harvested, viably frozen, and labeled as passage 0 (P0). To establish replicative senescent cells, MEFs were seeded at 3 × 10^5^ cells per 10 cm plate, trypsinized after 3 days, counted and reseeded at the same density from P0 to P10.

### 4.6 Biochemical assays

Plasma, hepatic, or cellular levels of triglycerides, total cholesterol, alanine transaminase (ALT), and aspartate transaminase (AST) were quantified using commercially available kits as previously described (Zhang et al., 2018). Blood SCAD concentrations and hepatic or cellular acetyl‐CoA levels were determined with commercial ELISA kits according to the manufacturer's instructions.

### 4.7 *Acads* gene overexpression and knockdown

*Acads* siRNA or scramble control siRNA was premixed with a dilution of Hieff Trans® liposomal transfection reagent in Opti‐DMEM and then incubated with HepG2 cells in FBS‐free DMEM for 6 h. After removal of the transfection media, the cells were cultured in fresh media for an additional 2 days. After 2 days of transfection, the HepG2 cells were treated with H_2_O_2_ with or without dichloroacetic acid (DCA, 12 μM) or ATP (10 μM) for 24 h and harvested for subsequent processing. For overexpression, HepG2 cells were transfected with an adenovirus encoding SCAD (Ad‐*Acads*).

### 4.8 Determination of ATP content

ATP levels were measured using a cellular ATP assay kit as previously described (Liu et al., 2021). Briefly, cells were lysed in 200 μL of cell lysis reagent, and the protein concentration was quantified with a BCA protein assay kit. After adding 1 μL of luciferase reagent and 100 μL of dilution buffer to 50 μL of lysate, the luminescence was analyzed after a 5‐s delay on a luminometer. The ATP content of the sample was calculated using a standard curve established from known levels of ATP. The results were adjusted to the sample's total protein content.

### 4.9 Histopathologic analysis and immunostaining

Liver tissue specimens were either embedded in Tissue OCT‐Freezing medium or fixed in 4% neutral buffered formalin (NBF) formaldehyde overnight followed by paraffin embedding. Five‐micron‐thick tissue sections were subjected to hematoxylin and eosin (H&E) staining, Masson's trichrome staining, Oil Red O staining, and BODIPY 493/503 staining as described previously (He et al., 2023; Yu et al., 2023).

SA‐β galactosidase staining was conducted using a SA‐β‐gal staining kit following the manufacturer's instructions. Briefly, frozen tissue sections were fixed with 2.5% glutaraldehyde solution followed by incubation with a staining solution (40 mM citric acid, 0.2 M Na_2_HPO_4_, 5 mM K_3_Fe(CN)_6_, 5 mM K_4_Fe(CN)6_3_H_2_O, 2 mM MgCl2, and 150 mM NaCl) and X‐Gal (1 mg/mL) at least overnight in a humid chamber at 37°C; thereafter, the samples were counterstained with 1% Fast Red for 2 min. Senescent cells were evaluated under a light microscope for the development of a blue color. For cellular SA‐β‐gal staining, after removing growth media, the cells were washed in 1× PBS, fixed with 4% formaldehyde, and stained with staining solution at least overnight at 37°C. Thereafter, the cells that were stained blue were counted under a light microscope.

Immunohistochemical analysis and immunostaining were conducted according to standard procedures using monoclonal antibodies against SCAD, LC3II, and p62/SQSTM1. Images were acquired using an Axio Imager A2 fluorescence microscope (Carl Zeiss, Germany).

### 4.10 Measurement of autophagy flux and lipophagy

Measurements of autophagic flux in H_2_O_2_‐induced senescent HepG2 cells were performed with an RFP‐GFP‐LC3 image‐based assay as reported previously (Singh et al., 2014). H_2_O_2_‐treated HepG2 cells with or without *ACADS* knockdown by siRNA were infected with a tandem‐tagged GFP‐RFP‐LC3 adenovirus. After 24 h of infection, the number of GFP‐LC3 (green)‐ and RFP‐LC3 (red)‐positive puncta were quantified by counting >50 cells on an inverted confocal microscope (Carl Zeiss, Germany) and were also measured by ratiometric flow cytometry (BD Biosciences, USA).

Lipophagy levels were measured as previously described (Yu et al., 2023). HepG2 cells treated with H_2_O_2_ or P10 MEFs were infected with RFP‐LC3 adenovirus. After 24 h of infection, the cells were stained with LysoTracker Red working solution and BODIPY 493/503. The colocalization of RFP‐LC3 with BODIPY 493/503 in cells was visualized and analyzed using a confocal microscope (Carl Zeiss, Germany).

### 4.11 Transmission electron microscopy (TEM)

TEM was performed as previously described (Liu et al., 2021). Briefly, mouse liver tissue specimens (<1 mm^3^) were fixed in 2.5% glutaraldehyde solution. After being postfixed in 1% OsO_4_, dehydrated and infiltrated with an increasing concentration gradient of ethanol and propylene oxide, the samples were embedded in propylene oxide/Poly/Bed 812 epoxy resin overnight. The solidified tissue specimens were cut into 50–60 nm slices and stained with 2% C_4_H_6_O_6_U and 1% C_6_H_8_O_7_Pb. Autophagosome‐like vesicles were observed under a Philips CM120 scanning transmission electron microscope.

### 4.12 RNA isolation, cDNA synthesis, and quantitative real‐time PCR (qRT–PCR)

Total hepatic or cellular RNA was extracted with TRIzol reagent and reversely transcribed into cDNA using a cDNA synthesis kit. qRT**–**PCR analysis with the indicated primers (Table S6) was carried out using SYBR Green technology as described previously (Liu et al., 2017). The relative mRNA levels were estimated by the comparative threshold cycle (ΔΔCt) method using Gapdh as a normalization vector.

### 4.13 Western blotting

Hepatic or cellular protein extraction, quantitation and western blotting were performed as described elsewhere. Briefly, 40 μg of protein per sample was separated via 8%–15% SDS**–**PAGE and subsequently transferred to polyvinylidene difluoride (PVDF) membranes. After blocking in skim milk for 1 h, the membranes were incubated with primary antibodies overnight at 4°C, followed by incubation with horseradish peroxidase (HRP)‐conjugated secondary antibodies. The protein bands were visualized using enhanced chemiluminescence (ECL) and imaged with a ChemiDoc gel system (Bio‐Rad), and the fold density was quantified using Quantity One software (Bio‐Rad Laboratories, Hercules, CA).

### 4.14 Statistical analysis

The data are presented as the mean ± standard deviation (SD). Differences between groups were tested using Student's *t* test and two‐way ANOVA. *p < 0.05, **p < 0.01, and ***p < 0.001 indicate statistical significance. Correlation analysis was performed using the Pearson's correlation test. These analyses were performed using GraphPad Prism software.

## AUTHOR CONTRIBUTIONS

DD, SSY, XQY, RXZ, YL, HMZ, DXC, XRF YTW, and XCQ conducted the experiments. ZGS wrote this article. ZGS is the guarantor of this work and takes full responsibility for the integrity of the data and the accuracy of the data analysis.

## CONFLICT OF INTEREST STATEMENT

The authors declare that there is no conflict of interest.

## Supporting information


Appendix S1.

## Data Availability

Three representative gene expression profile datasets (GES133815, GSE66676, GSE48452) were downloaded from the Gene Expression Omnibus (GEO) database (https://www.ncbi.nlm.nih.gov/geo/). Our previously available raw transcriptome data (GSE128608) were used to investigate statistically relevant biological associations via gene set enrichment analysis (GSEA) (https://www.gsea‐msigdb.org/gsea/index.jsp).
