# Supplementary material for: Aging‐induced short‐chain acyl‐CoA dehydrogenase promotes age‐related hepatic steatosis by suppressing lipophagy
Source: Aging Cell. 2024 Jun 19;23(10):e14256. doi: 10.1111/acel.14256 (PMC11464120; doi:10.1111/acel.14256)
Supplement: Supplementary file 1 — Appendix S1. [file ACEL-23-e14256-s001.docx]

**Aging-induced short-chain acyl-CoA dehydrogenase promotes age-related hepatic steatosis by suppressing lipophagy**

Dan Deng^†^, Shanshan Yang^†^, Xiaoqian Yu^†^, Ruixue Zhou, Yin Liu, Hongmei Zhang, Daxin Cui, Xingrong Feng, Yanting Wu, Xiaocun Qi, Zhiguang Su*

Molecular Medicine Research Center and National Clinical Research Center for Geriatrics, West China Hospital, and State Key Laboratory of Biotherapy, Sichuan University. Chengdu 610041, China.

**Figure S1**


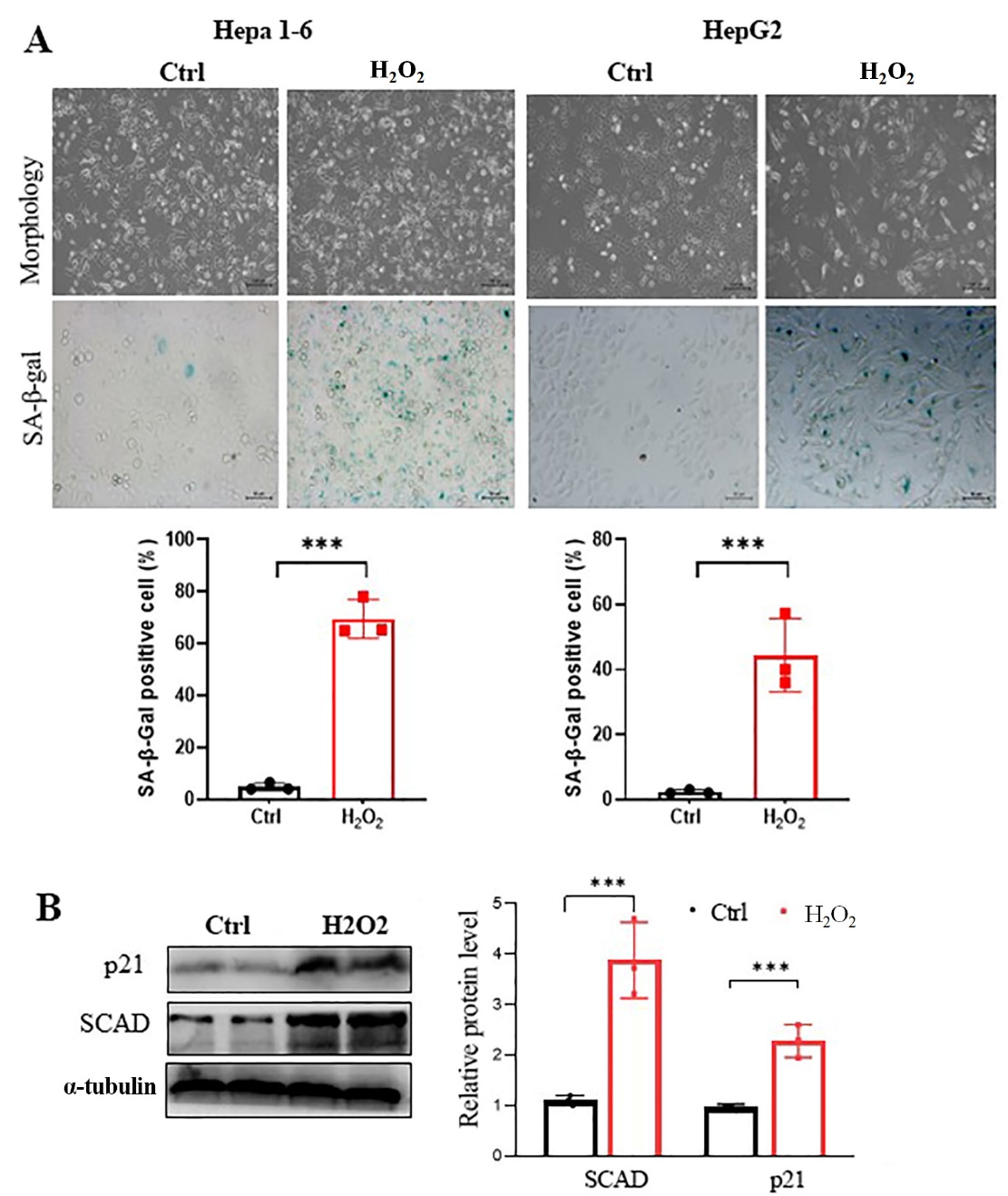


**Figure S1. SCAD levels increase in H_2_O_2_-induced senescent hepatocytes**. Mouse Hepa1-6 and human HepG2 hepatocytes were treated with H_2_O_2_ (0.4 mM for Hepa1-6 cells and 0.3 mM for HepG2 cells) for 4 consecutive days. (A) Senescence induction was assessed by cellular morphology (upper) and senescence‐associated (SA)‐β‐galactosidase staining (lower). (B) Protein levels of p21 and SCAD in Hepa1-6 cells were measured by immunoblotting using α-tubulin as loading control. Values were presented as the means ± S.D. of three independent experiments. ****p* < 0.001.

**Figure S2**


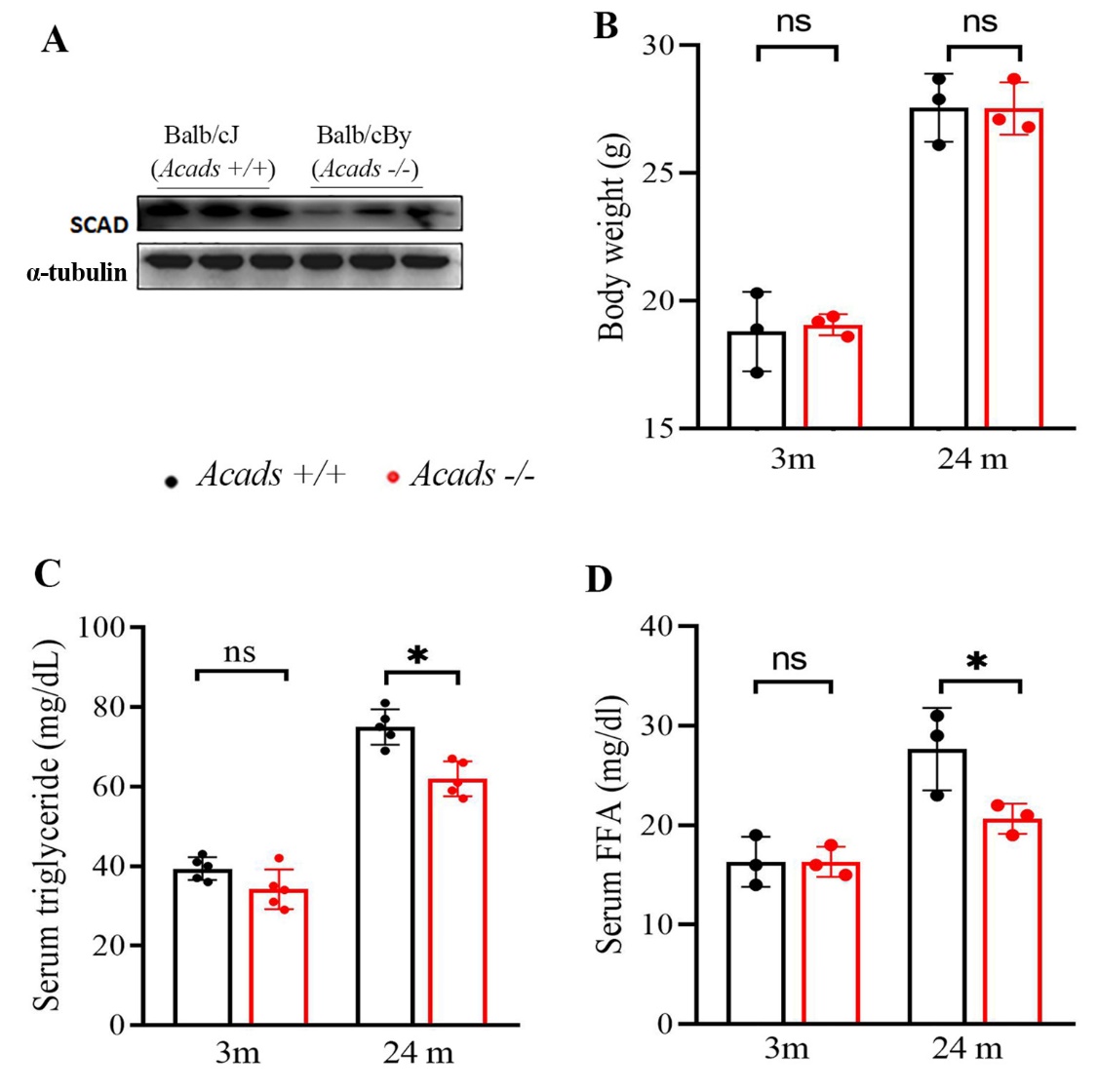


**Figure S2. *Acads* deficiency protects mice against age-related liver steatosis.** *Acads+/+* and *Acads-/-* mice were fed a chow diet. West blotting analysis measured SCAD levels in liver tissue (A), body weight (B) and serum triglycerides (C) and fatty acid (FA) concentrations (D) were measured when mice were 3 months (3 m) or 24 months (24 m) old. **P* < 0.05.

**Figure S3.**


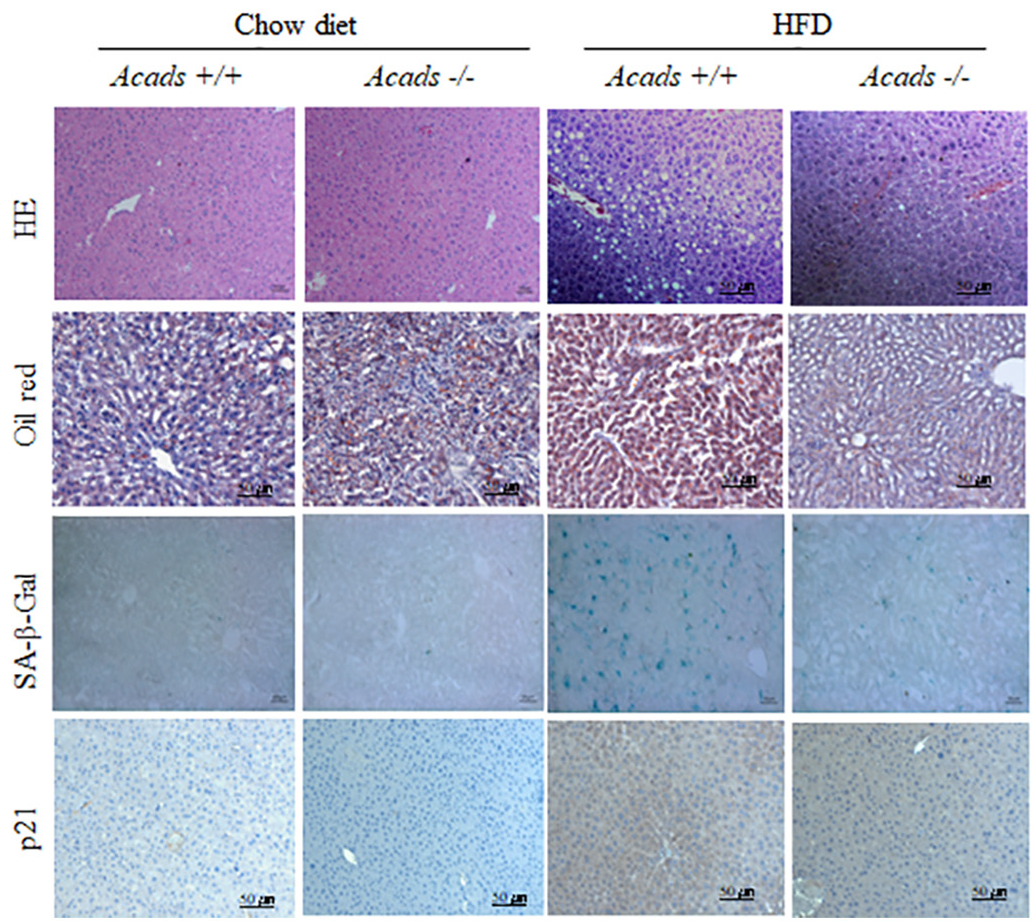


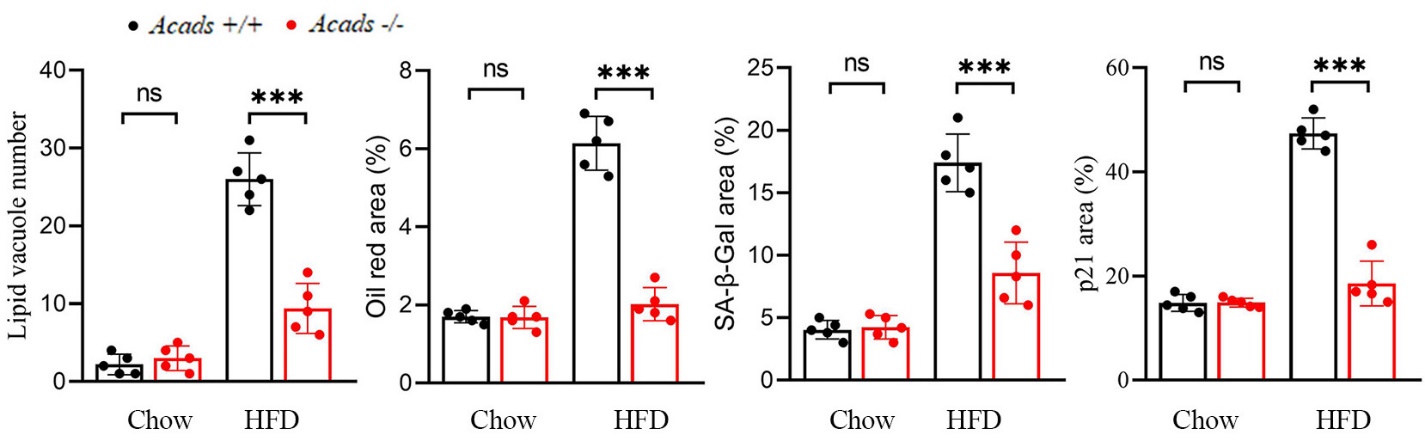


**Figure S3.** *Acads-/-* mice are resistant to diet-induced hepatic steatosis and liver aging. 8-wk-old *Acads+/+* and *Acads-/-* mice were fed a chow or an HFD for additionally consecutive 12 wk. Upper: representative images of liver sections stained with H&E, Oil Red O, senescence-associated (SA)-β-galactosidase, or p21 antibody (scale bar=50 μm). Lower: quantitative analysis of the number of lipid vacuoles in the liver and the areas of Oil Red O-, β-galactosidase-, or p21-positive staining. Three micrographs of each mouse were used for quantification (n = 5). ∗∗∗ *P* < 0.001 (unpaired Student's t test).

**Figure S4**


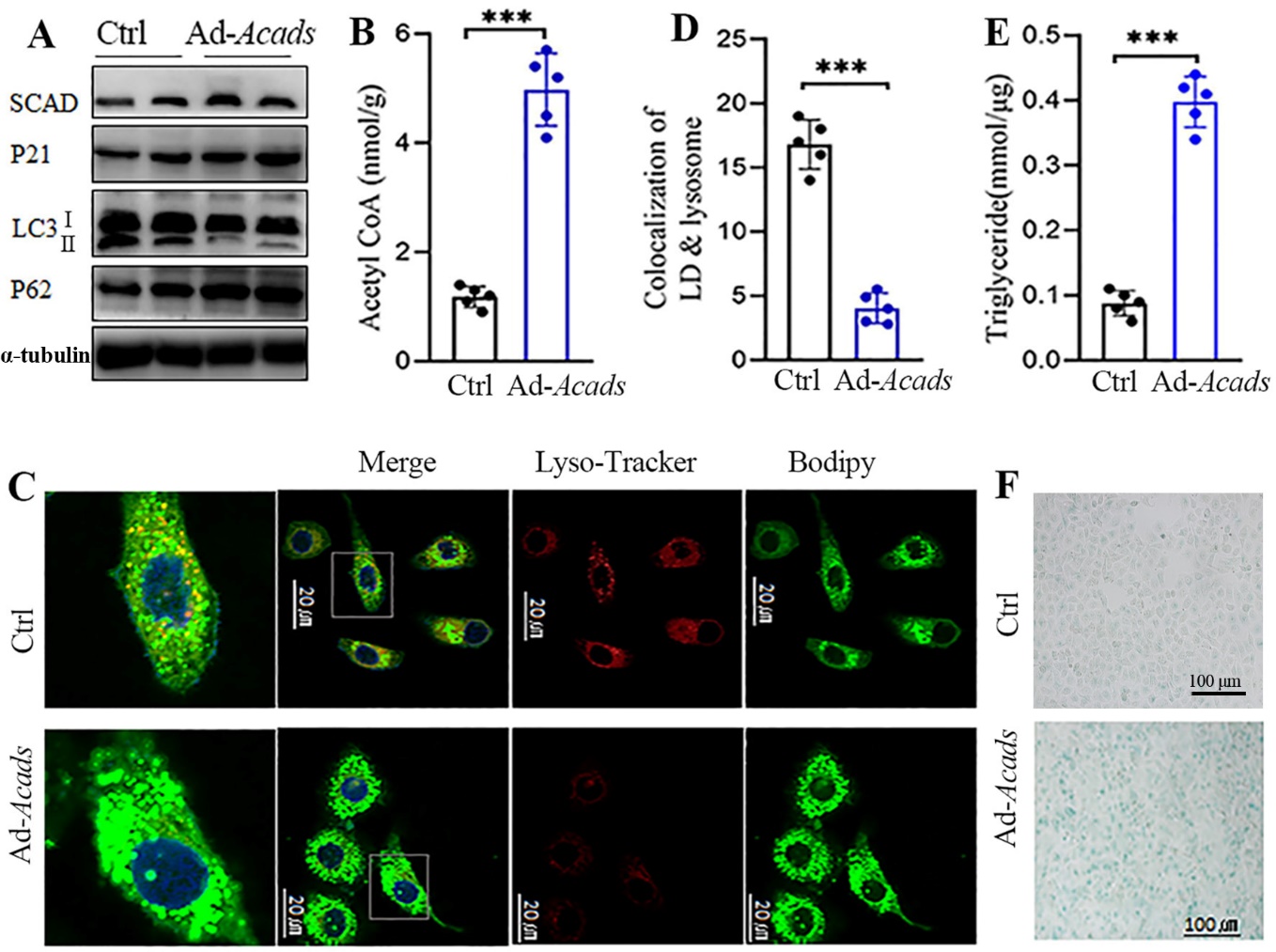


**Figure S4.** SCAD exacerbates senescence-associated liver steatosis through the acetyl-coenzyme A-dependent lipophagy pathway. HepG2 cells were transfected with an adenovirus-encoding SCAD (Ad-*Acads*). (A) Expression levels of SCAD, p21, LC3I/LC3II and p62 were assessed by Western blotting. (B) Cellular acetyl-CoA production was measured via ELISA. (C-D) Colocalization between lysosomes labeled with LysoTracker Red and lipid droplets labeled with the green lipid dye BODIPY was examined by confocal fluorescence microscope, representative images (scale bars = 20 μm) (C) and quantification of colocalization (D). (E) Cellular triglyceride content. (F) Representative image of SA-β-galactosidase staining. **p* < 0.05, ***p* < 0.01, and ****p* < 0.001.

**Supplementary Tables**

**Table S1**. GSEA pathways enriched in human liver tissues by aging

| Gene set name | NES | Nominal  *p*-value | FDR  *q*-value | |
| --- | --- | --- | --- | --- |
| ***Up regulated*** | | | |  |
| KEGG_INTESTINAL_IMMUNE_NETWORK_FOR_  IGA_PRODUCTION | 2.03 | 0.000 | 0.000 | |
| KEGG_LEISHMANIA_INFECTION | 1.99 | 0.000 | 0.045 | |
| KEGG_COMPLEMENT_AND_COAGULATION_ CASCADES | 1.88 | 0.000 | 0.039 | |
| KEGG_FATTY_ACID_METABLISM | 1.77 | 0.001 | 0.041 | |
| KEGG_BETA_ALANINE_METABOLISM | 1.76 | 0.003 | 0.076 | |
| KEGG_HISTIDINE_METABOLISM | 1.69 | 0.004 | 0.079 | |
| KEGG_GRAFT_VERSUS_HOST_DISEASE | 1.67 | 0.004 | 0.086 | |
| KEGG_CYTOKINE_CYTOKINE_RECEPTOR_ INTERACTION | 1.60 | 0.005 | 0.105 | |
| KEGG_HEMATOPOIETIC_CELL_LINEAGE | 1.58 | 0.006 | 0.165 | |
| KEGG_BUTANOATE_ METABLISM | 1.56 | 0.006 | 0.168 | |
| KEGG_DNA_REPLICATION | 1.53 | 0.008 | 0.175 | |
| KEGG_CELL_ADHESION_MOLECULES_CAMS | 1.47 | 0.018 | 0.188 | |
| KEGG_SYSTEMIC_LUPUS_ERYTHEMATOSUS | 1.33 | 0.001 | 0.203 | |
| ***Down regulated*** | | | |  |
| KEGG_HOMOLOGOUS_RECOMBINATION | -1.57 | 0.005 | 0.163 | |
| KEGG_CELL_CYCLE | -1.36 | 0.011 | 0.187 | |
| KEGG_GLIOMA | -1.24 | 0.022 | 0.219 | |
| KEGG_LONG_TERM_POTENTIATION | -1.19 | 0.021 | 0.238 | |
| KEGG_CHRONIC_MYELOID_LEUKEMIA | -1.09 | 0.025 | 0.245 | |

*Abbreviation:* GSEA: gene set enrichment analysis; NES: normalized enrichment score; FDR: false discovery rate.

Gene sets with NES > 1, nominal *p*-value <0.05 and FDR *q*-value <0.25 were considered as significant.

**Table S2**. Core genes associated with fatty acid metabolism

| Gene | | Gene | Gene | Gene | | |
| --- | --- | --- | --- | --- | --- | --- |
| *ACADS* | *ALDH2* | | *ACSL4* | | *ADH1C* |  |
| *CYP4A11* | *ACAA1* | | *HADH* | | *ADH6* |  |
| *ACADL* | *ALDH1B1* | | *ACAA2* | | *ADH1B* |  |
| *CPT1B* | *ACADM* | | *ADH4* | | *ECI2* |  |
| *ALDH3A2* | *ALDH7A1* | | *ECHS1* | | *ACSL3* |  |

**Table S3.** GSEA pathways enriched in mouse liver tissues by low expression of *Acads*

| Gene set name | NES | Nominal  *p*-value | FDR  *q*-value |
| --- | --- | --- | --- |
| ***Up regulated*** |  |  |  |
| KEGG_BASAL_CELL_CARCINOMA | 1.75 | 0.000 | 0.081 |
| KEGG_LYSOSOME | 1.64 | 0.001 | 0.092 |
| KEGG_GLYCOSYLPHOSOHATIDYLINOSITOL_  GPI_ANCHOR_BIOSYNTHESIS | 1.62 | 0.005 | 0.101 |
| KEGG_PYRIMIDINE_METABOLISM | 1.59 | 0.006 | 0.128 |
| KEGG_PROTEASOME | 1.58 | 0.006 | 0.133 |
| KEGG_GLUTATHIONE_METABOLISM | 1.55 | 0.007 | 0.169 |
| KEGG_REGULATION_OF_AUTOPHAGY | 1.07 | 0.009 | 0.195 |
| ***Down regulated*** |  |  |  |
| KEGG_BIOSYNTHESIS_OF_UNSATURATED_  FATTY_ACIDS | -1.56 | 0.001 | 0.128 |
| KEGG_BUTANOATE_METABOLISM | -1.48 | 0.030 | 0.176 |
| KEGG_BETA_ALANINE_METABOLISM | -1.42 | 0.049 | 0.195 |
| KEGG_FATTY_ACID_METABOLISM | -1.17 | 0.047 | 0.215 |

*Abbreviation*: GSEA: gene set enrichment analysis; NES: normalized enrichment score; FDR: false discovery rate.

Gene sets with NES > 1, nominal *p*-value <0.05 and FDR *q*-value <0.25 were considered as significant.

**Table S4**. Core genes associated with autophagy and lysosome

| Autophagy | Lysosome | | |
| --- | --- | --- | --- |
| IFNA8 | ACP2 | CTSK | GM2A |
| IFNA5 | ATP6V0A2 | CTSZ | ARSG |
| PRKAA1 | ACP5 | CTNS | HYAL1 |
| IFNA21 | AP4E1 | NAGA | PPT1 |
| ULK1 | MANBA | CTSS | GALNS |
| BECN1 | TPP1 | AP3D1 | CLTC |
| IFNA14 | CTSL | ASAH1 | LGMN |
| ULK3 | AGA | AP3M2 | SLC11A2 |
| IFNA1 | MAN2B1 | SORT1 | CLTB |
| ULK2 | AP1G1 | ATP6AP1 | GGA3 |
| ATG3 | CLN3 | ATP6V0C | GBA |
| IFNA4 | LAPTM5 | GLA | TCIRG1 |
| ATG4D | ATP6V0A1 | AP3S2 | CD68 |
| IFNA2 | CTSG | IGF2R | LAPTM4B |
| ATG4A | CTSA | CTSC | CD63 |
| IFNA10 |  |  |  |

**Table S5**. Clinical and biochemical characteristics of individuals with normal liver (NL)

and nonalcoholic fatty liver disease (NAFLD)

|  | NL (n=51) | NAFLD (n=68) |
| --- | --- | --- |
| Age (years) | 48.90 | 52.70 |
| BMI (kg/m^2^) | 21.40 | 29.60** |
| Male % | 53.90 | 58.30 |
| Female % | 46.10 | 41.70 |
| TG (mmol/L) | 1.23 | 3.43** |
| Glucose (mmol/L) | 5.11 | 5.83 |
| TC (mmol/L) | 4.63 | 6.73** |
| HDLc (mmol/L) | 1.68 | 1.02* |
| LDLc (mmol/L) | 1.97 | 3.19 |
| CREA (µmol/L) | 54.20 | 78.31*** |
| BUN (mmol/L) | 4.48 | 6.01 |
| URIC (mmol/L) | 324.31 | 548.21*** |
| ALT (IU/L) | 21.10 | 44.30** |
| AST (IU/L) | 19.20 | 33.60** |
| LPA (mg/L) | 173.21 | 683.71*** |

Data were expressed as the mean or percentage (%). Statistically significant differences were determined using Student's *t-*test and were denoted by **p* < 0.05, ***p* < 0.01, and ****p* < 0.001 when comparing NL versus NAFLD.

*Abbreviations*: ALT, alanine aminotransferase; AST, aspartate aminotransferase; BMI, body mass index; BUN, blood urea nitrogen; CHOL, cholesterol; CREA, creatinine; HDLc, high‐density lipoprotein cholesterol; LDLc, Low-density lipoprotein cholesterol, TG, triglyceride. URIC, uric acid.

**Table S6**. Sequences of primers for real time qPCR and siRNA

| Gene | Forward (5' to 3') | Reverse (5' to 3') |
| --- | --- | --- |
| *Scad* | TGGCGACGGTTACACACTG | GTAGGCCAGGTAATCCAAGCC |
| *Mcad* | GAAAGTTGCGGTGGCCTTGG | AAGCACACATCATTGGCTGGC |
| *Lcad* | GGGAAGAGCAAGCGTACTCC | TCTGTCATGGCTATGGCACC |
| *Vlcad* | CTACTGTGCTTCAGGGACAAC | CAAAGGACTTCGATTCTGCCC |
| *Acads siRNA* | CCACCGGAAUCUGAACCACUGCAUU | |
| Scramble siRNA | UAAGGCUAUGAAGAGAUA | |
